# Data on the effect of vertical agrivoltaic systems on the crop performance of oat (*Avena Sativa* L.) cultivated in Sweden

**DOI:** 10.1016/j.dib.2026.112984

**Published:** 2026-06-18

**Authors:** Simone Coluccia, Giovanni Urso, Davide Farruggia, Mario Licata, Torsten Hörndahl, Giuseppe Langella, Michelina Ruocco, Tekai Eddine Khalil Zidane, Arash Khosravi, Pietro Elia Campana

**Affiliations:** a Department of Biological Sciences, University of Naples Federico II, Naples 80126, Italy; b Institute for Sustainable Plant Protection (IPSP), National Research Council of Italy (CNR), Portici 80055, Italy; c Department of Agricultural, Food and Forest Sciences, University of Palermo, 90128 Palermo, Italy; d Greenable s.r.l., 90144 Palermo, Italy; e Research Consortium for the Development of Innovative Agro-Environmental Systems, 90143 Palermo, Italy; f Department of Biosystems and Technology, Swedish University of Agricultural Sciences, Alnarp, Sweden; g Department of Industrial Engineering, University of Naples Federico II, 80125 Naples, Italy; h Department of Engineering Sciences, Mälardalen University, 72123 Västerås, Sweden

**Keywords:** Oat yield, Leaf area index, Vertical agrivoltaics system, Ground-mounted photovoltaics system

## Abstract

Integrating solar panels with crops in the same piece of land is possible using agrivoltaic (AV) systems. However, AV systems alter the microclimate and present several challenges to crop performance; therefore, site-specific crop development monitoring and yield assessment are crucial. In this context, a field experiment was conducted in 2024 to investigate the growth and crop performance of oat cultivated under a vertical AV system and open-field reference condition at Kärrbo Prästgård near Västerås, Sweden. The dataset presented in this article considers two different system designs, vertical AV and conventional ground-mounted photovoltaic (CGMPV) systems. The biophysical parameter leaf area index was used to monitor oat development throughout the growing season. At harvest, both quantitative and qualitative yield performance were assessed using kernel and straw yields, thousand-kernel weight, and kernel crude protein and fat content.

All collected data were analysed using analysis of variance (ANOVA) with Tukey pairwise comparisons. It was observed that there was a significant variation in yield within the AV system. However, the mean total biomass (kernel and straw) in the AV system was higher than in both the reference and CGMPV systems; while the difference was not statistically significant compared to the reference system, it was significantly higher than that observed in the CGMPV system. Researchers can use this field-based data to better understand oat performance under the AV design and to perform further analyses or comparisons with yields from other locations, climatic zones, or system configurations. In addition, this empirical dataset can support the development and validation of crop models under similar conditions.

Specifications Table

**Table:** 

Subject	Earth & Environmental Sciences
Specific subject area	Agriculture and Solar Photovoltaic
Type of data	Figures and Tables. Analysed mean and row data.
Data collection	Data on oat (*Avena sativa* L.) cultivated at Kärrbo Prästgård (59.55° N, 16.76° E), Sweden, was collected during the growth period (from May 28 to September 2, 2024). Leaf area index (unitless) was measured throughout the growing season. At harvest on September 2, sampling was conducted in an area of 0.25 m² at each sampling point, and data were collected with five replications, totalling fifty-five samples. The collected data were statistically analysed as described in this paper.
Data source location	Mälardalen University, Sweden, is the owner of the data presented in this article. The field trail performed at Kärrbo Prästgård (59.55° N, 16.76° E), and altitude of 21 meters above sea level.
Data accessibility	Repository name: Zenodo Data identification number: 10.5281/zenodo.19594989 Direct URL to data: All data are on Zenodo and currently under restricted access, as the article has not yet been published. The direct links are provided in a separate document that has been submitted. Instructions for accessing these data: None
Related research article	None

## 1 Value of the Data

• The dataset captures the impact of vertical AV and CGMPV systems on oat cultivation in Sweden, measuring both yield and quality traits such as crude protein and fat content. By comparing oats grown under this AV configuration with those grown in open-field and CGMPV conditions, the data highlight how different system designs affect crop performance in a Nordic context.
• This dataset provides important information on oat development during the growing season under vertical AV system and CGMPV in Sweden, by employing the biophysical parameter of LAI. It captures growth trend over time at various points within the vertical AV system and highlights differences compared with conventional open field and CGMPV conditions.
• These data can be reused by researchers to evaluate and validate crop performance, perform comparative analyses across regions, and explore new AV configurations. By providing an accurate methodology for planting, growth monitoring, and sampling, as outlined in this study, the approach can be replicated for crop experiments under similar or different AV systems.

These results provide valuable information for developing models that assess crop responses to shading, guide sustainable AV management, and support decision-making for farmers, energy planners, and policymakers.

## 2 Background

The co-location of photovoltaic (PV) panels with agricultural land, known as AV, makes renewable energy conversion possible while sustaining agricultural activity [1,2]. However, it presents several technical, agronomic, and economic challenges. Key technical considerations include system configuration, module height, row spacing, and orientation [3]. Agronomic challenges include reduced Photosynthetically Active Radiation (PAR), microclimate alterations, and crop suitability, while economic concerns include land–energy trade-offs, profitability, and policy incentives [[3], [4], [5]]. Several studies have focused on optimizing AV system designs, yet findings indicate that crop performance under AV installations is highly dependent on local climate conditions, panel orientation, and structural configuration [5,6].

This dataset explores oat (*Avena sativa* L.) performance under a vertical AV system and a CGMPV system in Sweden. Such data are critical for farmers, policymakers, and investors seeking to evaluate the feasibility of dual land use, plan incentives, or make informed crop and system choices. Seasonal field trials, coupled with validated modeling tools, are necessary to predict site potential and optimize AV performance, ensuring reliable integration of PV systems with sustainable agriculture [2,7]. To the best of our knowledge, this is one of the few studies to present empirical data on oat development and yield under an AV system in northern latitudes. Among the studies most closely related to our work, Urso et al. [8] investigated oat cultivation in a vertical AV system in Sweden. They used a methodology similar to ours regarding sampling points within the vertical AV system and mainly focused on detecting photosynthesis trends. Although they also presented quantitative crop performance data in terms of total dry matter biomass yield (kernel + straw, kg ha⁻¹) and showed the variation between different locations within the vertical AV system, our study differs in several aspects. In our work, we present both quantitative and qualitative crop performance data in a way that highlights spatial variation within the vertical AV system while also comparing total production. Additionally, we present data on CGMPV, which was not investigated in their study. In another related study, Campana et al. [9], who developed an optimization algorithm for a vertical AV system in Sweden by combining a bifacial PV model with two crops, including oat. The work focused only on modelling and simulation and was validated against county-level statistics.

## 3 Data Description

**Fig. 1:**
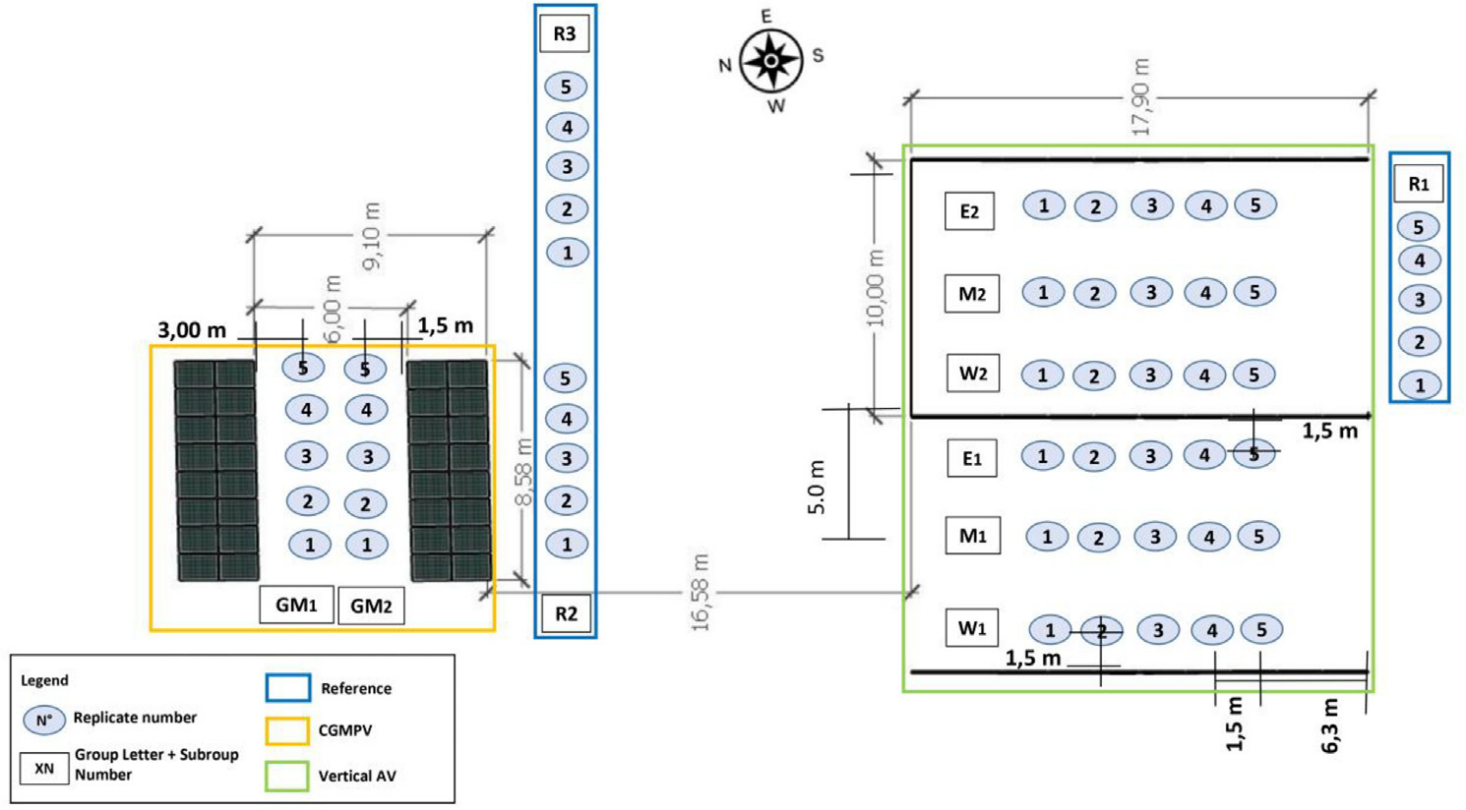
Top view of the crop experiment layout showing oat grown in a vertical AV system, a CGMPV system, and reference plots, located at Kärrbo Prästgård, Sweden. Schematic representation not drawn to scale.

In 2024, an experimental trial on oats was carried out at Kärrbo Prästgård (59.55° N, 16.76° E), Sweden, succeeding a barley trial conducted on the same plot the previous year [7]. The dataset includes agronomic measurements and statistical analyses of key quantitative and qualitative parameters, including LAI (unitless), yield in Dry Matter (DM) (kg/ha) for kernels and straw, Thousand Kernel Weight (TKW) (g), and crude protein and fat content as a percentage of Dry Weight (% of DW). The statistical analysis was conducted on the five distinct groups (Fig. 1).

1. W groups: Sampling points on the west portion of the vertical AV system rows.
2. M groups: Sampling points on the middle portion of the vertical AV system rows.
3. E groups: Sampling points on the east portion of the vertical AV system rows.
4. GM groups: Sampling points in the CGMPV system.
5. R groups: Sampling points in the open field, reference system

**Fig. 2:**
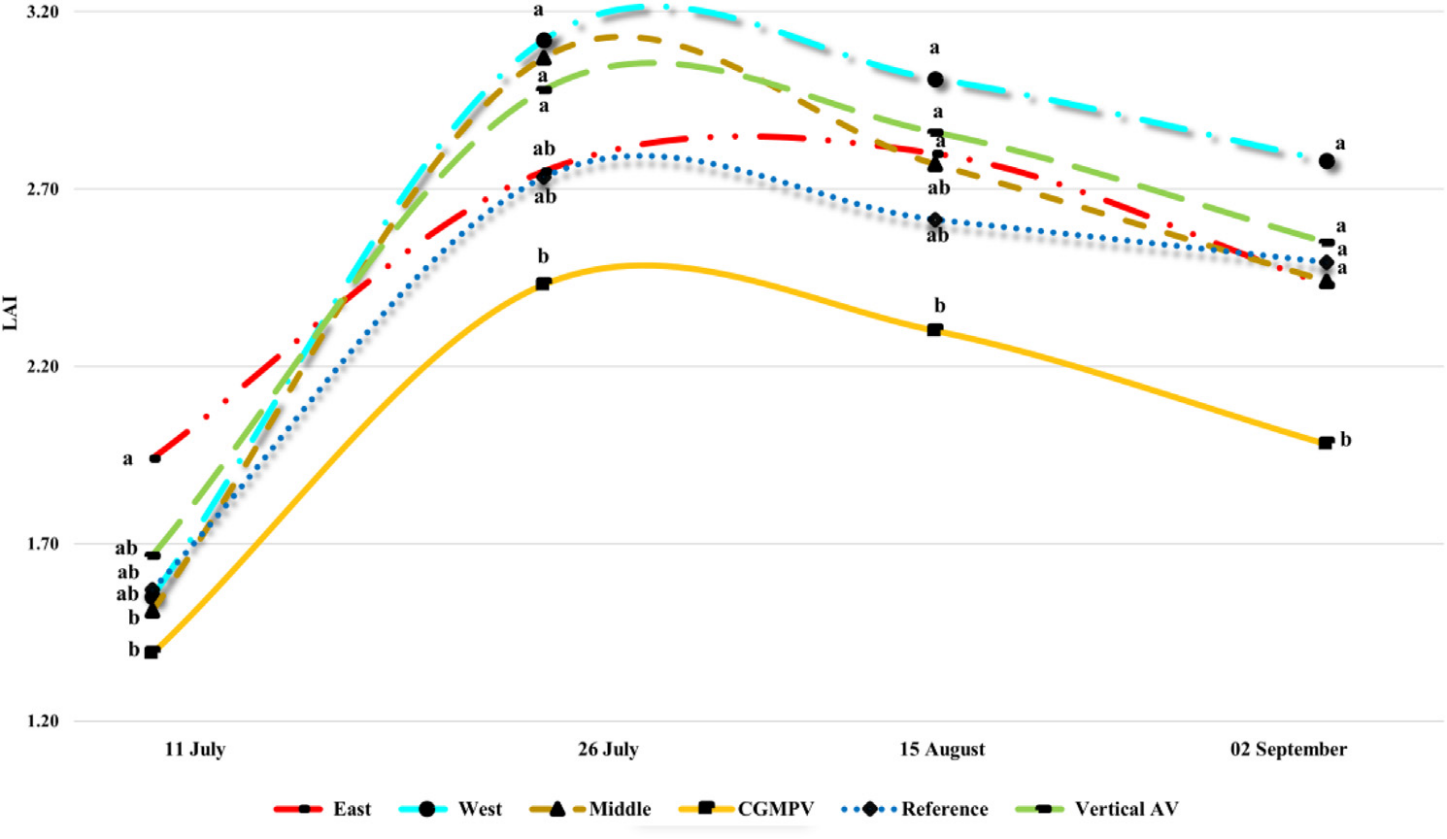
Pattern of oat Leaf Area Index (LAI) variation across sampling groups on four measurement dates (July 11 and 26, August 15, and September 2). Data analysed using one-way ANOVA with Tukey pairwise comparison with a 5% (p < 0.05) significance. Different letters indicate significant differences.

LAI trends during the experimental period at four measurement dates (July 11 and 26, August 15, and September 2) were analysed using one-way ANOVA with Tukey pairwise comparisons at a 5% significance level (Fig. 2). The W group exhibited significantly higher average LAI values across all measurement dates, while the GM group consistently showed the lowest average values throughout the survey periods.

The statistical analysis for the yield of kernel DM (kg/ha) shows significant differences between the groups (Table 1). The one-way ANOVA with Tukey pairwise comparison with a 5% significance showed that group M had the highest average yield compared to all other groups, while the groups W and E showed a higher average production than R but not statistically significant; the same groups are statistically significant compared to the GM group, which had the lowest average yield.

**Table 1:** Statistical analysis of oat kernel yield DM (kg/ha) cultivated at Kärrbo Prästgård in 2024, using one-way ANOVA with Tukey pairwise comparisons at a 5% significance level (p < 0.05). Different letters indicate significant differences between groups. No land loss attributable to the PV panel structure was included in the calculation of kernel yield per hectare for the AV groups (W, M, E) and GM.

Groups	Mean	Median	SD	Min	Max	L. Quantile	U. Quantile
W	3506^b^	3429	473	2835	4339	3185	3874
M	4435^a^	4509	615	3233	5171	3997	4993
E	3228^b^	3351	546	2304	4092	2744	3642
R	2874^b^	2540	882	1912	4453	2063	3980
GM	1769^c^	1593	483	1342	2921	1493	1963

Statistically significant differences (p < 0.05) between groups were observed in straw DM (Kg/ha), as reported in Table 2. One-way ANOVA with Tukey pairwise comparison indicated that group M had the highest mean, while GM had the lowest.

**Table 2:** Statistical analysis of oat straw yield DM (kg/ha) cultivated at Kärrbo Prästgård in 2024, using one-way ANOVA with Tukey pairwise comparisons at a 5% significance level (p < 0.05). Different letters indicate significant differences between groups. No land loss attributable to the PV panel structure was included in the calculation of kernel yield per hectare for the AV groups (W, M, E) and GM.

Groups	Mean	Median	SD	Min	Max	L. Quantile	U. Quantile
W	3669^ab^	3813	375	2863	4069	3404	3940
M	4302^a^	4411	630	2795	5006	4105	4745
E	3139 ^bc^	3130	197	2853	3399	2951	3344
R	3889^ab^	3783	841	2372	5465	3244	4617
GM	2466^c^	2264	855	1569	3813	1677	3392

In terms of total biomass (kernel + straw) DM (kg/ha), Table 3 shows marked differences between group means. The Tukey test at a 5% significance level indicates that group M, significantly different from the other groups, produced 50% more than the GM group, which had the lowest yield. Groups W, E, and R were also significantly higher than GM.

**Table 3:** Statistical analysis of oat total biomass (kernel + straw) yield DM (kg/ha) cultivated at Kärrbo Prästgård in 2024, using one-way ANOVA with Tukey pairwise comparisons at a 5% significance level (p < 0.05). Different letters indicate significant differences between groups. No land loss attributable to the PV panel structure was included in the calculation of kernel yield per hectare for the AV groups (W, M, E) and GM.

Groups	Mean	Median	SD	Min	Max	L. Quantile	U. Quantile
W	7175^b^	7227	682	5699	8008	6776	7819
M	8737^a^	8961	1061	6870	10078	7872	9581
E	6367^b^	6324	600	5623	7472	5852	6909
R	6763^b^	6299	1615	5026	9445	5161	8615
GM	4235^c^	3696	1222	3081	6734	3353	5098

For TKW (g), one-way ANOVA with Tukey pairwise comparisons (p < 0.05) revealed statistically significant differences only between group E and the other groups, as summarized in Table 4.

**Table 4:** Statistical analysis of oat TKW (g) cultivated at Kärrbo Prästgård in 2024, using one-way ANOVA with Tukey pairwise comparisons at a 5% significance level (p < 0.05). Different letters indicate significant differences between groups.

Groups	Mean	Median	SD	Min	Max	L. Quantile	U. Quantile
W	39.3^a^	38.9	1.5	37.3	41.6	38.1	40.8
M	39.8^a^	39.8	0.8	38.5	40.9	39.1	40.4
E	35.3^b^	35.5	0.7	34.1	36.1	34.7	35.8
R	38.3^a^	38.2	1.9	35.1	42.9	36.9	39.4
GM	38.5^a^	38.6	1.1	37.0	40.5	37.4	39.4

Regarding crude protein (% of DW), group W obtained the highest average, while group E obtained the lowest average, as shown in Table 5.

**Table 5:** Statistical analysis of oat crude protein (% of DW) cultivated at Kärrbo Prästgård in 2024, using one-way ANOVA with Tukey pairwise comparisons at a 5% significance level (p < 0.05). Different letters indicate significant differences between groups.

Groups	Mean	Median	SD	Max	Min	L. Quantile	U. Quantile
W	10.9^a^	10.9	0.2	11.3	10.6	10.7	11.1
M	10.4^bc^	10.4	0.3	11.0	10.1	10.2	10.6
E	10.3^c^	10.3	0.3	10.8	9.9	10.0	10.5
R	10.7^ab^	10.7	0.3	11.2	10.2	10.5	10.8
GM	10.4^bc^	10.3	0.3	10.8	9.9	10.2	10.5

For crude fat (% of DW), the group W demonstrated the highest mean, and the group E the lowest, as can be seen in Table 6.

**Table 6:** Statistical analysis of oat crude fa (% of DW) cultivated at Kärrbo Prästgård in 2024, using one-way ANOVA with Tukey pairwise comparisons at a 5% significance level (p < 0.05). Different letters indicate significant differences between groups.

Groups	Mean	Median	SD	Max	Min	L. Quantile	U. Quantile
W	5.3^b^	5.4	0.2	5.6	5.1	5.2	5.5
M	5.6^a^	5.7	0.2	5.9	5.4	5.5	5.8
E	5.5^ab^	5.5	0.1	5.7	5.3	5.4	5.6
R	5.6^a^	5.6	0.2	6.0	5.3	5.5	5.7
GM	5.4^b^	5.4	0.2	5.6	5.0	5.2	5.5

**Fig. 3:**
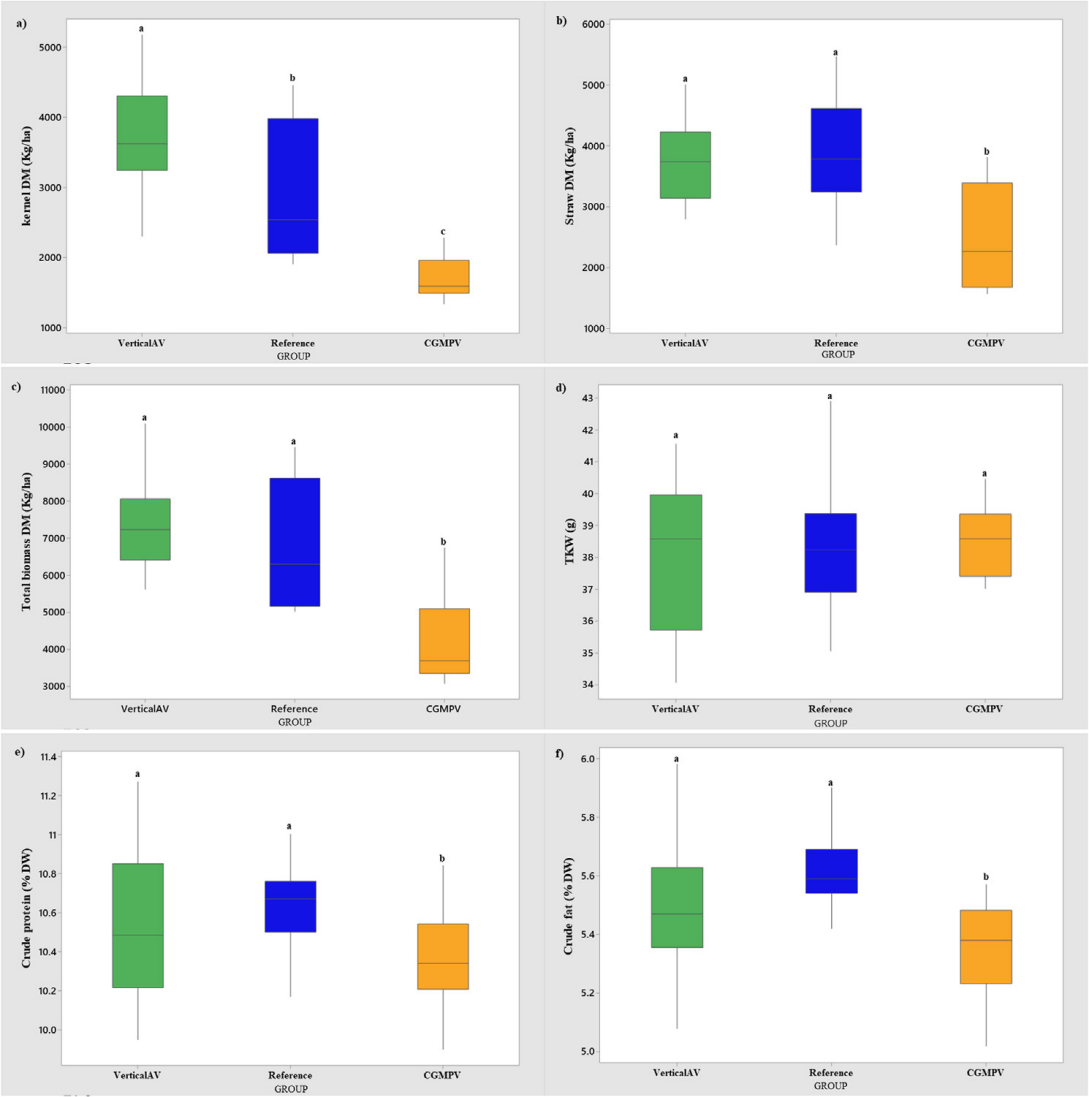
Oat performance (yield and quality) cultivated under the AV system, CGMPV system, and Reference at Kärrbo Prästgård in 2024. a) Kernel DM (kg/ha); b) Straw DM (kg/ha); c) Total biomass (kernel + straw) DM (kg/ha); d) TKW (g); e) Crude protein (% DW); f) Crude fat (% DW). Statistical analysis was performed using one-way ANOVA with Tukey pairwise comparisons at 5% significance (p < 0.05). For panels a–c, a 10% land loss due to the PV panel structure was included in the calculation for the Vertical AV.

Statical analysis performed using one-way ANOVA with Tukey pairwise comparison at a 5% significance level (p < 0.05) for the three blocks (vertical, ground-mounted and Reference) showed significant differences between the blocks, as presented in fig. 3.

For Figs. 3a, 3b, 3c, Fig. 3, Fig. 3 the vertical one was statistically higher than the ground-mounted group. For Fig. 3d, the vertical one had a higher mean than ground-mounted, but the difference was not statistically significant. A similar trend was observed for the Reference compared to the ground-mounted block.

## 4 Experimental Design, Materials and Methods

### 4.1 Agrivoltaic system description

AV system located in the Kärbbo Prästgård (59.55° N, 16.76° E), which is also known as the first agrivoltaic site in Sweden [7]. The vertical AV system is oriented in an almost north–south direction and consists of 60 bifacial PV modules (Jolywood JW-D72N-380, Jolywood (Taizhou) Solar Technology Co., Ltd., China), with an installed capacity of 22.8 kWp. It is arranged in three rows with a length of 17.9 m and a row-to-row distance of 10 m. The CGMPV system consists of 32 bifacial PV modules (LONGi LR4-60HBD-370M, LONGi Green Energy Technology Co., Ltd., China), with an installed capacity of 11.8 kWp. It is arranged in two rows with a length of 8.6 m and a row-to-row distance of 9.1 m. An overview of the main characteristics of the vertical AV and CGMPV systems is provided in Table 7.

**Table 7:** An overview of the main characteristics of the vertical AV and CGMPV systems

	Vertical AV	CGMPV
Azimuth angle (°)	-84	6
Tilt angle (°)	90	30
Power (kWp)	22.8	11.8
Number of strings	2	2
Row-to-row distance (m)	10	9.1
PV module length (m)	1.974	1.755
PV module Width (m)	0.992	1.038
PV module efficiency (%)	19.4	20.3
PV module temperature coefficient of max power (%/°C)	-0.38	-0.35

### 4.2 Experimental layout

On May 28, 2024, under rainfed conditions, oats (*Avena sativa* L. variety Delfin Havre) were cultivated at a seeding density of 220 kg/ha. A fertiliser consisting of 50 kg of “Biofer” pelletized [10] with a N-P-K content of 10-3-1 was distributed simultaneously. No other agronomic practices were carried out.

Considering the soil characteristics of the study area, the soil exhibited slightly acidic conditions, with an average pH of 5.9. The mean available phosphorus, potassium, and magnesium contents were 7.6, 26.2, and 45.3 mg/100 g soil, respectively. The average potassium-to-magnesium ratio was 0.6, indicating a balanced nutrient relationship.

The soil texture was classified as clay loam, consisting on average of 30.8% clay, 48.3% silt, and 14.5% sand. The mean organic matter content was 6.4%. Furthermore, the average total carbon, nitrogen, and calcium concentrations were 36.8, 3.3, and 5.8 g/kg, respectively. Overall, the soil conditions across the study area were relatively homogeneous and moderately fertile.

To evaluate the differences among the three blocks of vertical AV, CGMPV, and the Reference, five groups were established. The vertical AV block was divided into three groups: W in the west, M in the middle, and E in the east. Each group further divided into two subgroups: W1 and W2, M1 and M2, and E1 and E2. The CGMPV block consisted of a single group organised into two subgroups, GM1 and GM2. The Reference block was a single group divided into three subgroups, R1, R2, and R3 (Fig. 1).

Each subgroup contained five replicates, yielding a total of 55 representative samples. The decision to partition the areas (W, M, and E) among the PV panels in the vertical AVs was made to conduct a more in-depth analysis of growth dynamics within each section. This division enables us to investigate how the variations in irradiation and shading impact plant growth dynamics. The data relating to the AV plant, from an engineering and energy perspective, are contained in the work carried out by Campana et al. [2].

### 4.3 Weather data collection

The climate data were gathered directly from the field weather station, where hourly measurements of the following parameters were recorded: air temperature (°C), relative humidity (%), and precipitation (mm/h).

For each day, the average temperature, relative humidity, vapor pressure deficit (VPD) (kPa), and precipitation were computed. Monthly averages and maximum and minimum values were determined for temperature, relative humidity, and VPD. VPD was calculated using the following equation [11]:

VPD=(1−(RH/100))×SVP

$$\text{SVP}=610.7\times 10^{\left(7.5T/237.3+T\right)}$$

In which RH (%) stands for relative humidity, SVP is the saturated vapor pressure (kPa), and T(°C) is the temperature.

VPD attained its peak average in June at 0.96 kPa, subsequently declining to its lowest point of 0.23 kPa in September. The mean air temperature fluctuated from 13.63 ±2.68°C in May to 18.06 ±3.37°C in June, with varying extremes recorded between 4.5°C and 27.5°C. Furthermore, relative humidity exhibited a progressive increase throughout the growing season, rising from an average of 51.58±9.99 % in May to 87.56 ±6.67% in September. Notably, maximum humidity levels approached 100% during August and September (Table 8).

**Table 8:** Monthly variation in VPD (kPa), air temperature (°C), and relative humidity (%) during the experimental period (from May to September). For each parameter, the mean ± SD, minimum, and maximum values are reported.

Month	VPD (kPa)			Temperature (°C)			Relative humidity (%)		
	Mean	Min	Max	Average	Min	Max	Average	Min	Max
May	0.77±0.20	0.16	0.99	13.63±2.68	-0.60	23.30	51.58±9.99	22.70	90.10
June	0.96±0.30	0.33	1.52	18.06±3.37	4.50	27.50	53.93±12.34	18.00	95.30
July	0.46±0.18	0.13	0.85	16.49±1.66	9.20	24.70	76.05±7.77	41.30	98.10
August	0.25±0.11	0.06	0.54	16.19±1.51	9.20	23.70	87.00±5.63	47.80	99.70
September	0.23±0.13	0.07	0.42	16.01±2.01	8.50	22.30	87.56±6.67	51.40	100.00

**Fig. 4:**
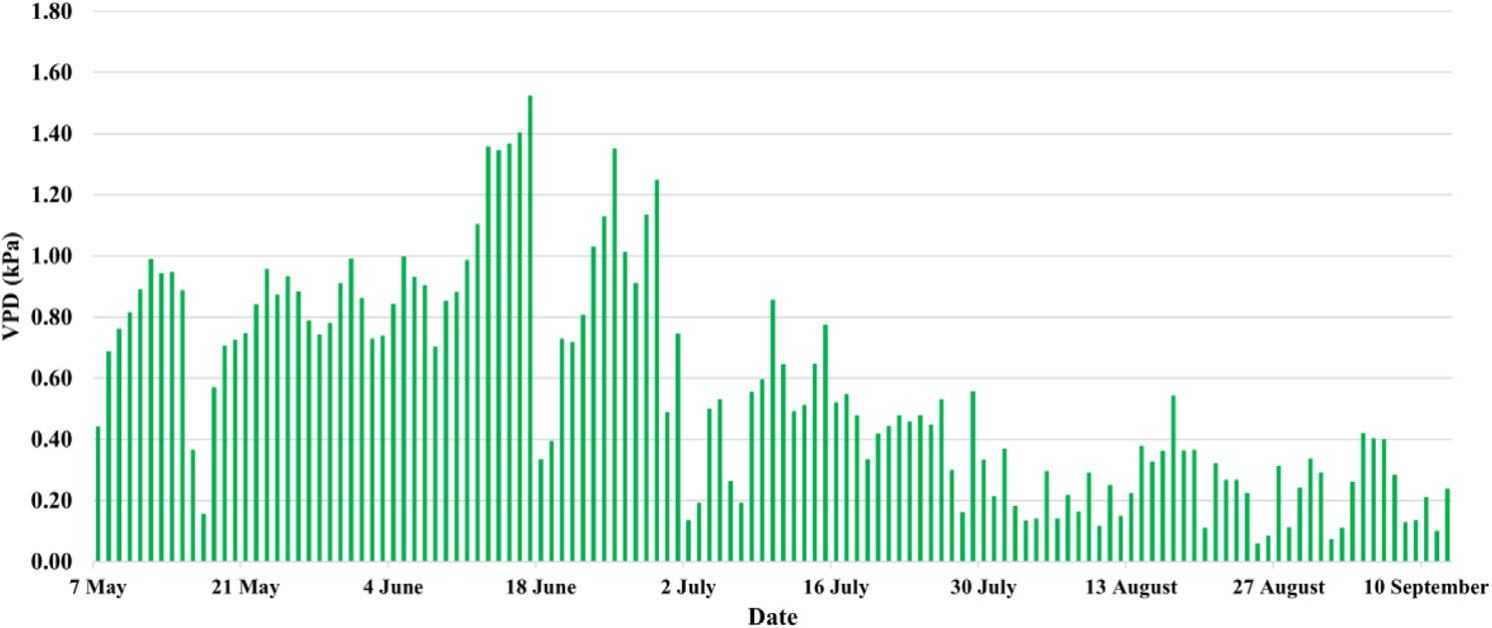
Trend of the VPD (kPa) during the experimental period (from May 7 to September 12).

Higher values were observed in May and June, reaching peaks exceeding 1.4±0.30 kPa, in mid-to-late June. Starting in July, a distinct downward trend emerged, with daily averages consistently falling below 0.6±0.11 kPa throughout September (Fig. 4). This seasonal pattern indicates a gradual increase in atmospheric humidity, as observed also in the climatic data presented in Table 7.

**Fig. 5:**
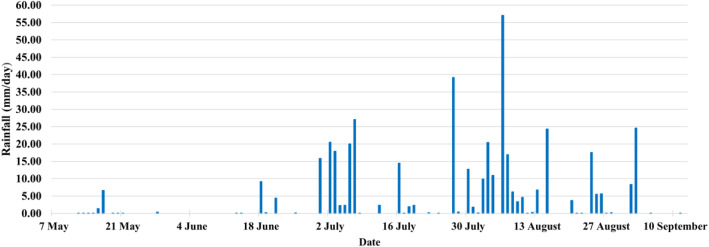
Daily rainfall (mm/day) during the experimental period (from May 7 to September 12) recorded by the weather station located in the field.

From July onwards, there was a significant increase in both frequency and intensity. The highest peak of rainfall was recorded on August 6, exceeding 55 mm/day, with further heavy precipitation occurring between July and August, with totals exceeding 25 mm/day. Overall, precipitation exhibited substantial intra-seasonal variability, which may have impacted crop development (Fig. 5).

### 4.4 Leaf Area Index

During the experiment, the LAI was measured and recorded at four distinct times (July 11 and 26, August 15, and September 2) using the SunScan canopy analysis system (SS1 model, Delta-T Devices Ltd., Cambridge, United Kingdom). According to the use manual, an ellipsoidal leaf angle distribution parameter (ELADP) value of 0.9 was adopted for all measurements. The data for the west and middle sections of the vertical AV block were collected in the morning. In contrast, those referring to the east section were acquired in the afternoon, under conditions without shading provoked by the AV structure, to avoid interference with the measured parameters.

### 4.5 Crop data collection

On September 2, when the oat was fully mature, samples were manually harvested. Based on the experimental layout described in Fig. 1 and in the section “experimental design, materials and methods” section, each sample within every subgroup was collected from a 0.25 m² area marked using custom-built metal frame [8]. The samples were cut approximately 5 cm above ground level. In total of 55 samples were harvested and considered for further analysis. All weeds were then removed from the collected samples, leaving only the kernels and straw.

### 4.6 Yield of kernel and straw

Immediately after harvest, the fresh weight of the samples (kernel + straw) was measured in the field using an accurate balance (Wellish, model YP20002, accuracy 0.01 g). The harvested samples were then transported to the laboratory and dried in an oven at 60°C for 72 hours. After drying, the samples were weighed to determine the dry matter (DM), considering both the total biomass (kernel + straw) and each component separately.

### 4.7 Thousand kernel weight

The TKW measurement was conducted using a special OPTO-AGRI (Opto Machines) machine, which employs image processing techniques to count the number of kernels in the sample and determine their weight [7].

### 4.8 Nutrient content analysis

The technique used to quantify the moisture, protein, and fat content of the kernels is specified in the European Standard EN 15948:2010. This technique uses Near-Infrared Transmittance (NIT) combined with an Artificial Neural Network (ANN) prediction model and an associated database. Through using the analyser (Infratec 1242 FOSS), the moisture, protein, and fat content of the kernel were determined [7]. The Swedish Food Agency has approved the use of this calibrated model for large-scale food analysis.

### 4.9 Data analysis

The statistical analysis was performed using one-way ANOVA with Tukey's pairwise comparisons at the 5% significance level (p < 0.05). It was performed using Minitab® 19 Statistical Software.

## Limitations

Weather conditions can significantly affect crop growth, development, and yield. Under current climate change scenarios, shifts in a region’s climate can move conditions outside the optimal temperature range for crop performance and create less stable weather patterns, posing challenges for agricultural management [12]. In such cases, long-term experiments conducted over consecutive years are necessary to collect more reliable data. Such long-term monitoring is currently ongoing at the study site; however, yearly results are also important, as the industry requires timely data to support the development and management of AV systems, where ensuring sufficient crop yield under PV panels is a key challenge [7,8,13,14]. Local conditions, including soil properties, nutrient availability, water supply, and agronomic management practices, may affect actual crop responses under AV shading [4]. Another limitation of this study is the relatively low number of samples and replicates. This constraint is common in agronomic experiments involving AV systems, where spatial and technical limitations, particularly in non-commercial setups, restrict replication [7,8].

## CRediT Author Statement

**Simone Coluccia:** Writing – original draft, Formal analysis, Visualization. **Giovanni Urso:** Writing – review & editing, Formal analysis. **Davide Farruggia:** Writing – review & editing. **Mario Licata:** Writing – review & editing. **Torsten Hörndahl:** Writing – review & editing, Methodology. **Giuseppe Langella:** Writing – review & editing. **Michelina Ruocco:** Writing – review & editing. **Tekai Eddine Khalil Zidane:** Writing – review & editing, Investigation. **Arash Khosravi:** Writing – original draft, Formal analysis, Methodology, Investigation, Conceptualization, Supervision. **Pietro Elia Campana:** Writing – review & editing, Resources, Funding acquisition, Conceptualization, Supervision.

## Ethics Statement

The dataset collected in this study did not involve animals, humans, or any data collected from social media platforms.

## Data Availability

ZenodoCrop performance - Kärrbo- 224 (Original data)
